# Microcystic Adnexal Carcinoma: A Rare and Locally Aggressive Adnexal Neoplasm

**DOI:** 10.7759/cureus.113537

**Published:** 2026-07-28

**Authors:** Diana Maria Ibarra Tostado, Teresa Jannete Ortega Valls, Renata Monserrat Espejo Nuño, Alexia Maria Lemus Núñez, José Alfredo Soto-Ortiz

**Affiliations:** 1 Dermatology, Instituto Dermatológico de Jalisco "Dr. José Barba Rubio", Guadalajara, MEX; 2 Internal Medicine, Instituto Mexicano del Seguro Social, Guadalajara, MEX; 3 Dermatology, Tecnológico de Monterrey Campus Guadalajara, Zapopan, MEX

**Keywords:** cutaneous adnexal neoplasms, dermato-oncologic surgery, histopathology examination, microcystic adnexal carcinoma, mohs micrographic surgery

## Abstract

Microcystic adnexal carcinoma (MAC) is a rare, locally aggressive cutaneous neoplasm with a high potential for local recurrence. It primarily affects the head and neck region and is thought to arise from pluripotent adnexal keratinocytes. The tumor is characterized by slow, progressive growth. A 70-year-old man presented with a lesion in the right supraorbital region, consisting of an irregular, yellowish, firm, and poorly defined plaque. A punch biopsy revealed an infiltrative neoplasm composed of nests and cords of atypical basaloid cells. The patient underwent Mohs micrographic surgery followed by reconstruction using an advancement flap incorporating superior and inferior Burow's triangles. The postoperative course was uneventful, with no evidence of recurrence after six months of follow-up. MAC is a rare cutaneous neoplasm that is frequently underdiagnosed and has a high risk of local recurrence if not completely excised. Mohs micrographic surgery is the treatment of choice because it allows complete margin assessment while maximizing tissue preservation.

## Introduction

Microcystic adnexal carcinoma (MAC) is a rare cutaneous adnexal neoplasm arising from skin appendages, including sweat glands and hair follicles, characterized by locally aggressive behavior and a high potential for local recurrence. It was first described in 1982, and since then, approximately 2,000 cases have been reported in the literature [1,2]. MAC most commonly arises in the head and neck region, particularly the upper lip. It is believed to originate from pluripotent adnexal keratinocytes, as it demonstrates eccrine, apocrine, follicular, and sebaceous differentiation [3,4].

Owing to its slow-growing nature, subtle clinical presentation, and frequent resemblance to benign adnexal tumors or basal cell carcinoma (BCC), MAC is often underrecognized, which may result in delayed diagnosis or misclassification. Accurate recognition of MAC is essential, as incomplete excision may be associated with a significant risk of local recurrence and prolonged disease progression. Therefore, appropriate diagnosis and surgical management are critical to optimize therapeutic outcomes. Mohs micrographic surgery (MMS) is widely considered the preferred surgical approach for MAC, particularly in anatomically sensitive areas, because it provides optimal margin control while preserving healthy tissue [4].

This case highlights the management of MAC in a supraorbital location using MMS followed by reconstruction with an advancement flap incorporating Burow’s triangles, providing an example of an effective approach to achieve both oncologic control and favorable functional and esthetic outcomes.

## Case presentation

**Figure 1:**
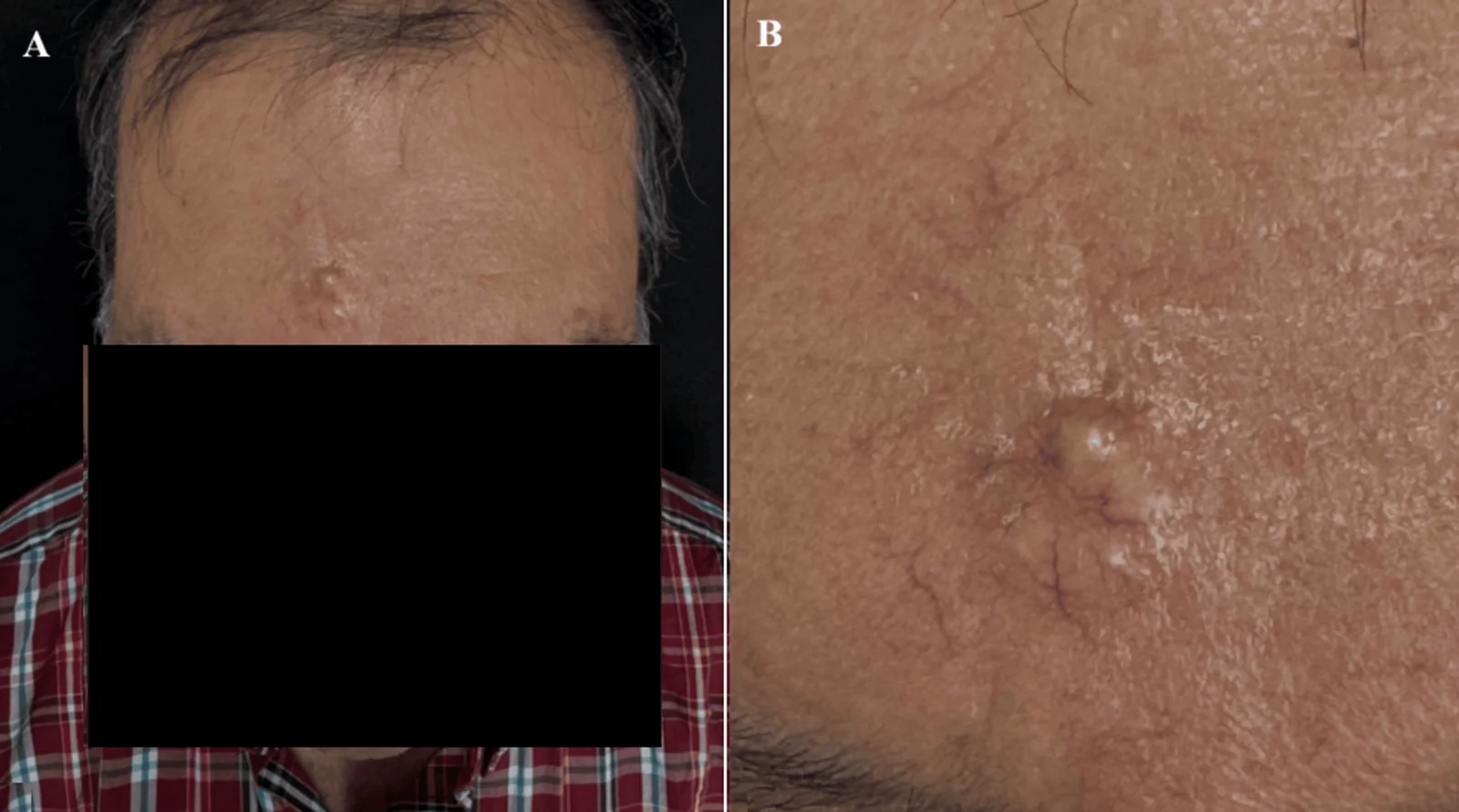
Clinical presentation. (A) Neoplasm located in the right supraorbital region. (B) Irregular, yellowish neoplasm measuring 1 × 1 × 0.4 cm, with focal areas of elevation, superficial telangiectatic vessels, poorly defined borders, a firm consistency, and an infiltrated, non-tender appearance on palpation.

A 70-year-old man with a medical history significant only for well-controlled type 2 diabetes mellitus presented with a lesion on the forehead, specifically in the right supraorbital region. Physical examination revealed an irregular, yellowish neoplasm measuring 1 × 1 × 0.4 cm, with focal areas of elevation, superficial telangiectatic vessels, poorly defined borders, a firm consistency, and an infiltrated, non-tender appearance. The lesion had been present for approximately six months and was asymptomatic, with slow progressive growth (Figure 1).

**Figure 2:**
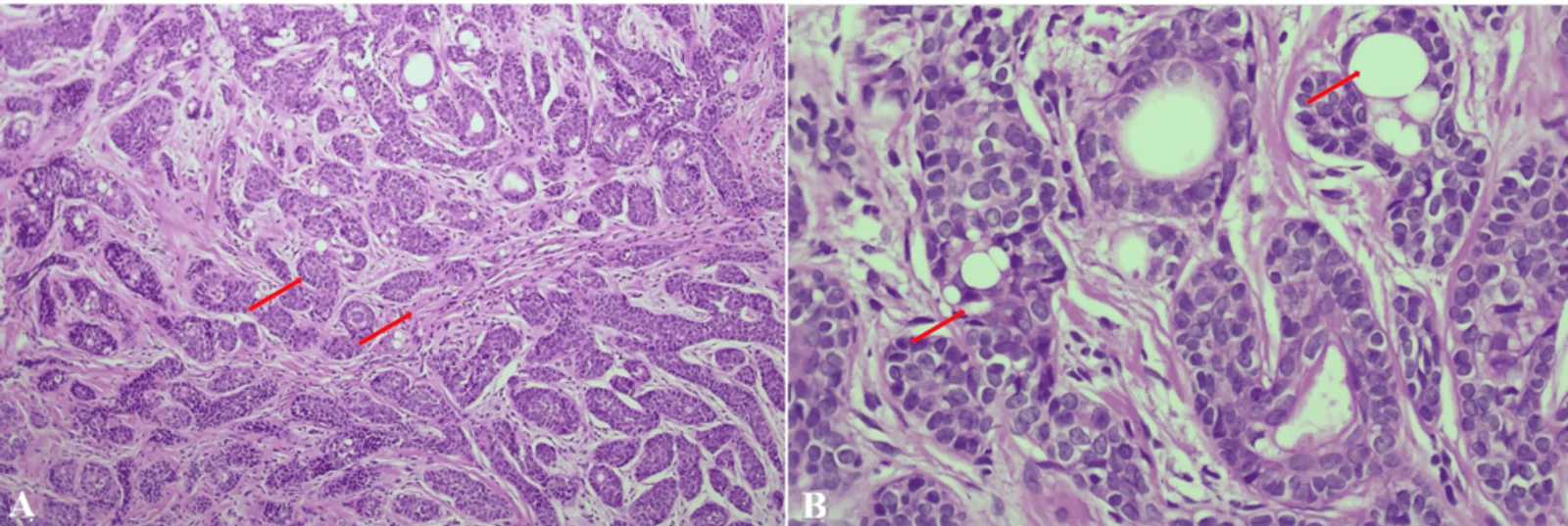
Histopathologic findings. (A) H&E, 40×. Nests and cords of basaloid cells embedded within a desmoplastic stroma are observed. (B) H&E, 100×. Tubular and cystic structures distributed throughout the dermis are identified. H&E: hematoxylin and eosin.

A punch biopsy specimen was obtained, revealing an infiltrative dermal epithelial neoplasm composed of numerous nests, cords, and ductal and microcystic structures extending throughout the reticular dermis. The neoplastic cells were predominantly basaloid, exhibiting mild cytologic atypia and focal intracytoplasmic vacuolization. Neither atypical mitotic figures nor tumor necrosis was identified. These histopathologic findings were consistent with MAC. Immunohistochemical studies were not performed because the diagnosis was considered sufficiently supported by the characteristic histopathologic features observed on routine hematoxylin and eosin staining (Figure 2).

**Figure 3:**
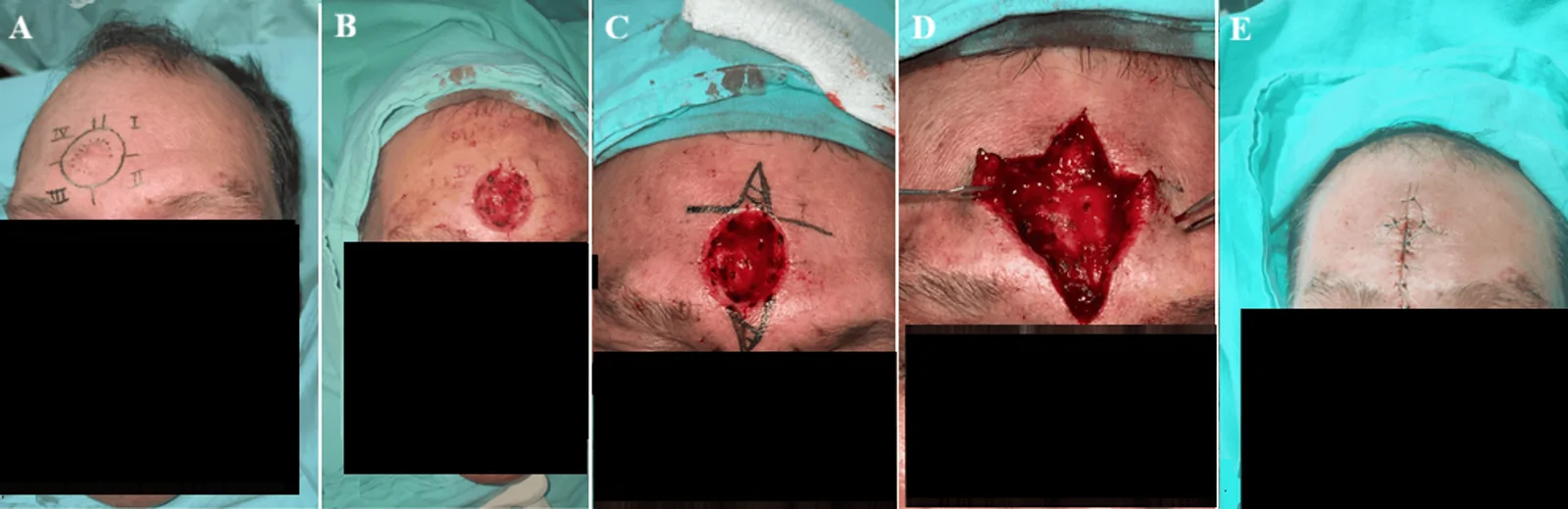
Surgical management. (A) Preoperative marking of resection margins for MMS. (B) Surgical defect following tumor excision. (C) Advancement flap design incorporating superior and inferior Burow's triangles. (D) Advancement flap prior to mobilization and closure. (E) Immediate postoperative result following closure with the advancement flap. MMS: Mohs micrographic surgery.

The patient subsequently underwent MMS, achieving complete tumor clearance with histologically negative margins after the second Mohs stage. The resulting surgical defect was reconstructed using an advancement flap incorporating superior and inferior Burow's triangles (Figure 3).

**Figure 4:**
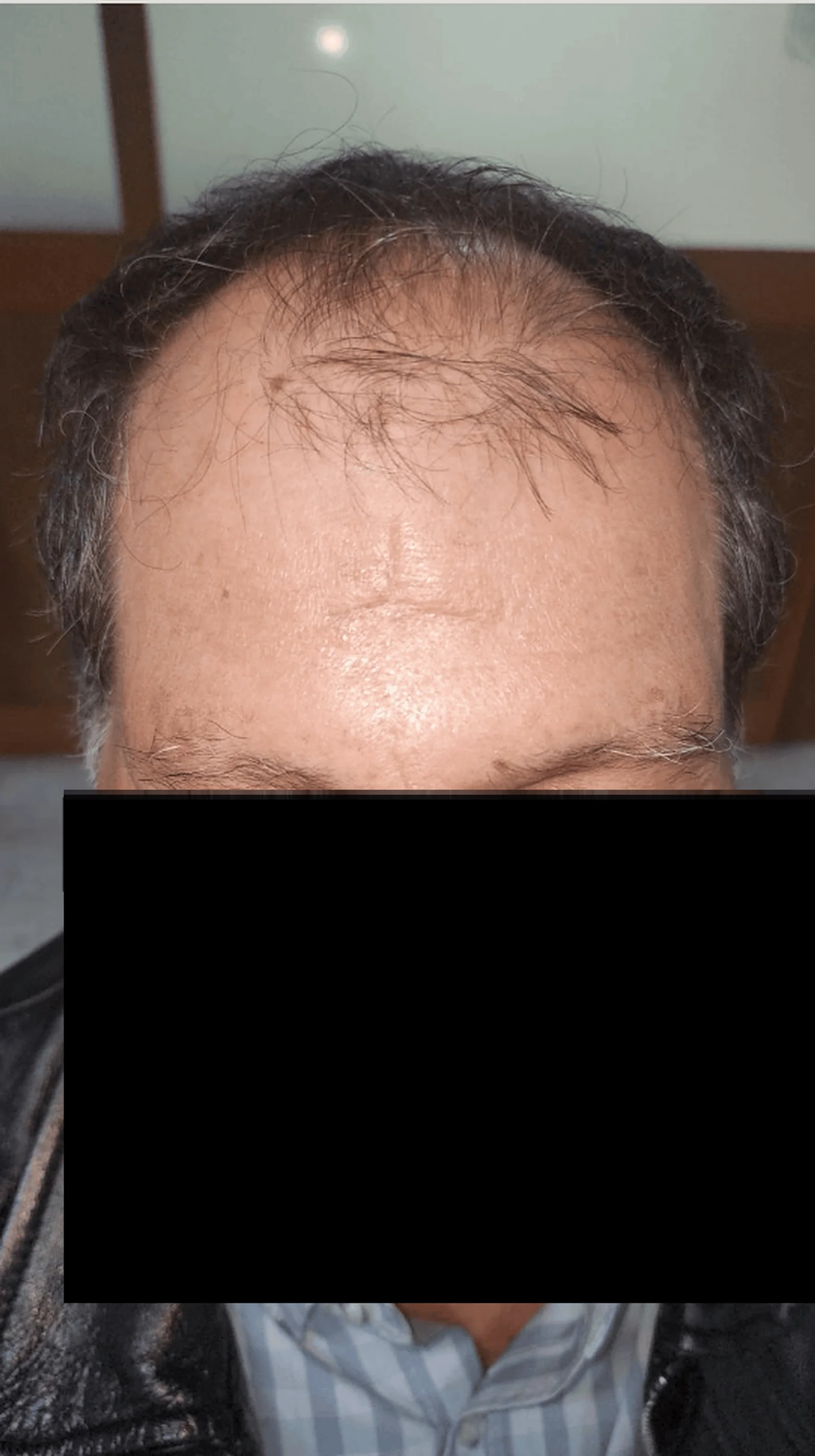
Postoperative outcome at six-month follow-up, showing no evidence of recurrence and a satisfactory esthetic result.

The postoperative course was uneventful, with satisfactory functional and cosmetic outcomes. At six months of follow-up, there was no clinical evidence of local recurrence (Figure 4).

## Discussion

Most reported cases of MAC have occurred in Caucasian patients, with a mean age at diagnosis of 61.8 years and a slight female predominance. MAC is an uncommon neoplasm, with an estimated incidence ranging from 1.6 to 6.5 cases per 10 million individuals [1,2,5].

Proposed risk factors include chronic ultraviolet radiation exposure, immunosuppression, and a history of therapeutic irradiation. Clinically, MAC typically presents as a poorly circumscribed plaque, nodule, or cystic lesion that is skin-colored, erythematous, or yellowish in appearance. It most frequently involves the head and neck region and is characteristically asymptomatic, firm on palpation, and slowly progressive [2-5]. Additional clinical findings may include a pearly border, ulceration, cutaneous atrophy, and superficial telangiectasias [3].

Perineural invasion has been reported in fewer than 5% of primary tumors; however, it has been documented in up to 87.5% of recurrent lesions, highlighting its association with locally advanced disease and recurrence. Rare cases of MAC with orbital involvement and intracranial extension have also been described. To date, no definitive association has been established between tumor size or anatomical location and the risk of local recurrence [6,7].

The main differential diagnoses include desmoplastic trichoepithelioma and BCC, which can be distinguished through histopathologic examination and immunohistochemical evaluation [2]. In this case, the diagnosis of MAC was supported by infiltrative cords and nests of basaloid cells with ductal differentiation and perineural invasion, features that are uncommon or absent in desmoplastic trichoepithelioma and BCC [3,6].

Histologically, MAC is characterized by epidermal sparing and involvement of the papillary dermis and superficial reticular dermis. Within the dermis, small keratin-filled cystic structures with infundibular and isthmic follicular differentiation are identified. The mid and deep reticular dermis contains multiple infiltrative nests and cords of basaloid cells exhibiting intracytoplasmic vacuolization. Focal ductal differentiation may be present, characterized by well-formed lumina lined by one or two layers of flattened epithelial cells. The neoplasm frequently extends beyond the clinically apparent margins, infiltrating the subcutaneous tissue, perineural spaces, and, in advanced cases, skeletal muscle [2,6].

Punch or excisional biopsies are recommended to establish an accurate histopathologic diagnosis, as the specimen should include adequate depth and subcutaneous tissue. Superficial biopsies are discouraged because they are associated with a diagnostic error rate of approximately 27% [1,7]. However, in this case, a punch biopsy was considered appropriate due to the size and anatomical location of the lesion, which allowed for adequate sampling of the tumor. The obtained specimen included the characteristic histopathologic features necessary for diagnosis, allowing for the correct identification of MAC. Furthermore, the cytologic features of MAC are often subtle, with minimal atypia and absent or scarce mitotic activity. These characteristics may lead to misinterpretation as benign adnexal neoplasms, such as syringoma or trichoepithelioma, particularly in facial lesions [1,7].

When routine histopathologic findings are inconclusive, immunohistochemistry may provide valuable diagnostic support. MAC typically demonstrates immunoreactivity for CK17, CK15, carcinoembryonic antigen (CEA), epithelial membrane antigen (EMA), CK5/6, and p63 [7].

Recent transcriptomic studies have identified upregulation of four genes involved in calcium signaling pathways: *CACNA1S*, *ATP2A1*, *RYR1*, and *MYLK3*. These genes exhibit increased RNA and protein expression compared with both normal and neoplastic sweat glands, suggesting their potential utility as novel diagnostic biomarkers and therapeutic targets [1].

MMS is considered the treatment of choice for MAC. When MMS is unavailable, wide local excision with 1-2 cm margins, including the underlying subcutaneous tissue, is recommended. Owing to its tissue-sparing approach and comprehensive margin assessment, MMS offers superior local tumor control while maximizing functional and cosmetic outcomes, particularly for tumors arising in cosmetically sensitive areas of the head and neck. Reported recurrence rates following MMS range from 0% to 12%, compared with 40%-60% after conventional wide local excision [2].

Radiotherapy has also been proposed as an adjunctive therapeutic modality; however, it is not recommended as monotherapy because of concerns regarding the induction of a more aggressive histologic phenotype. Consequently, its use is generally reserved for selected patients with recurrent disease or adverse histopathologic features, including perineural, lymphovascular, or vascular invasion, or extension into skeletal muscle or bone. Distant metastasis is exceedingly rare, with an estimated incidence of approximately 0.2%; isolated cases involving the vertebrae and lungs have been reported. Post-treatment surveillance is recommended every 6-12 months during the first five years after definitive treatment [6,7]. Given the relatively short follow-up period in our patient and the potential for late local recurrence in MAC, continued close surveillance is planned. Annual follow-up should be maintained for up to 10 years to monitor for disease recurrence [7]. This case illustrates the importance of early recognition, accurate histopathologic diagnosis, and appropriate management with MMS, resulting in an excellent clinical outcome without evidence of recurrence.

## Conclusions

MAC is a rare cutaneous neoplasm characterized by slow growth but significant local infiltrative potential, and its definitive diagnosis requires histopathologic confirmation, as its clinical presentation may mimic other more common benign or malignant entities. This case highlights the importance of maintaining a high index of suspicion for lesions with a chronic course, poorly defined borders, and infiltrative growth, as well as the fundamental role of MMS as the treatment of choice, allowing for complete tumor excision with maximal preservation of healthy tissue and histologic margin control. Reconstruction with an advancement flap incorporating Burow's triangles proved to be an effective and safe technique for restoring function and aesthetics in this anatomical location. The absence of clinical recurrence on follow-up reinforces the value of this comprehensive therapeutic approach. However, given that MAC may recur several years after treatment, longer-term follow-up is required to confirm sustained disease control.
